# Crystal structure of (±)-[*trans*-cyclo­hexane-1,2-diylbis(aza­nedi­yl)]di­phospho­nium dibromide dichloro­methane disolvate

**DOI:** 10.1107/S2056989016004576

**Published:** 2016-03-31

**Authors:** Aurora Rodríguez Álvarez, Hugo Tlahuext, Jean-Michel Grévy

**Affiliations:** a Centro de Investigaciónes Químicas, Universidad Autónoma del Estado de Morelos, Av. Universidad No. 1001, Col. Chamilpa, CP 62209, Cuernavaca Mor., Mexico

**Keywords:** crystal structure, *trans*-di­amino­cyclo­hexa­ne, di­phospho­nium ligands, C—H⋯Br and C—H⋯π inter­actions

## Abstract

The cation of the title solvated salt, C_42_H_42_N_2_P_2_^2+^·2Br^−^·2CH_2_Cl_2_, lies on a crystallographic twofold rotation axis. The 1,2-di­amino­cyclo­hexane fragment has a chair conformation with two N atoms in a *transoid* conformation [N—C—C—N = 163.4 (2)°]. In the crystal, the cations are linked to the anions by N—H⋯Br and C—H⋯Br hydrogen bonds, forming a chain structure along the *c* axis. The di­chloro­methane mol­ecule takes part in the hydrogen-bond network through C—H⋯π and C—H⋯Br inter­actions.

## Abstract 2

The crystal structure of the title compound shows that the 1,2-di­amino­cyclo­hexane fragment has a chair conformation with the N atoms in an anti­periplanar conformation. The packing is stabilized *via* N—H⋯Br, C—H⋯Br and C—H⋯π inter­actions.

## Chemical context   

Quaternary phospho­nium salts are very attractive compounds possessing widespread applications in synthetic organic chemistry and have played various important roles as stoichiometric reagents, phase-transfer reagents, reactive inter­mediates, ionic liquids, building blocks for supra­molecular assemblies and catalysts (Werner, 2009 ▸). In particular, *P*,*P*,*P*-triaryl-*P*-amino­phospho­nium salts bearing a primary amino group are isolable inter­mediates in the Horner & Oediger (1959 ▸) synthesis of imino­phospho­ranes. The title phospho­nium compound was used to synthesize new chiral imino­phospho­rane complexes in view of its catalytic application for organic transformations including olefin-CO copolymerization (Tardif *et al.*, 1998 ▸) and enanti­oselective copper-catalysed cyclo­propanation (Reetz & Bohres, 1998 ▸), but its crystal structure had not been determined.
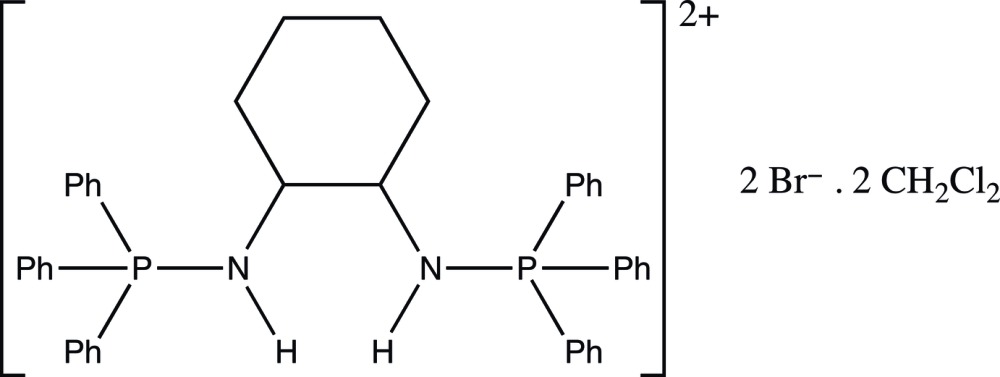


## Structural commentary   

**Figure 1:**
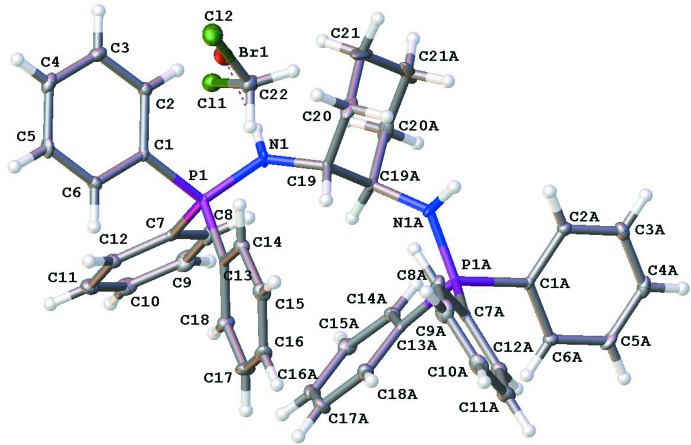
The mol­ecular structure of the title compound, showing the atom-labelling scheme. Atoms with the suffix A are at the symmetry position (−*x* + 1, *y*, −*z* + 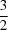).

The cation is situated on a crystallographic twofold rotation axis (Fig. 1 ▸). The 1,2-di­amino­cyclo­hexane fragment has a chair conformation with N atoms in a *transoid* conformation [N1—C19—C19^i^—N1^i^ = 163.4 (2)°; symmetry code: (i) −*x* + 1, *y*, −*z* + 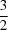]. The phospho­rus atom has a tetra­hedral geometry; the C—P—C angles are in the range 108.61 (12)–108.89 (12)° and the N—P—C angles in the range 109.47 (12)–111.00 (12)°. The N—P distance is 1.623 (2) Å.

## Supra­molecular features   

**Figure 2:**
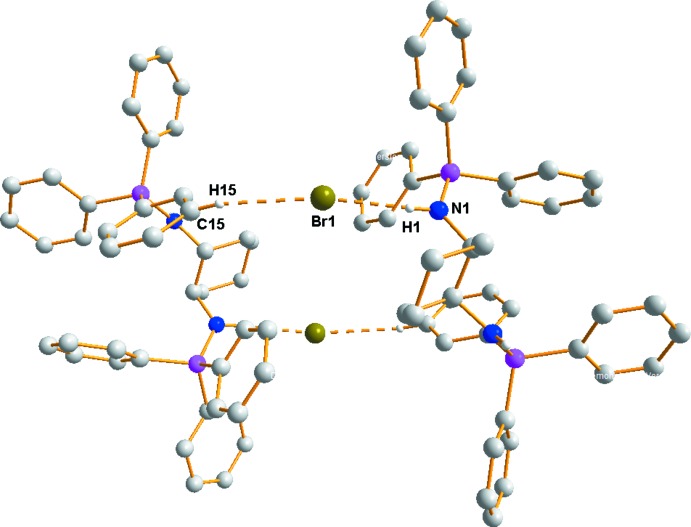
A cation dimer of the title compound formed by N—H⋯Br and C—H⋯Br hydrogen bonds (dashed lines) with a centrosymmetric 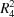
(22) motif.

**Figure 3:**
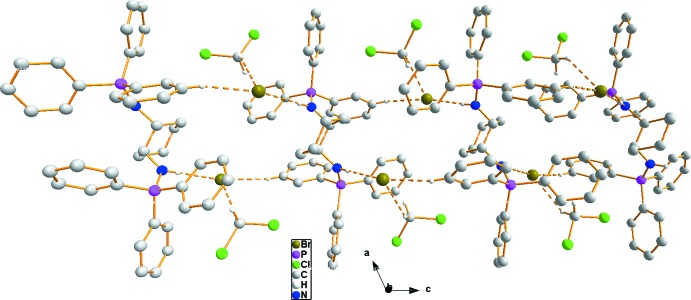
A view of the supra­molecular chain, generated by the N—H⋯Br and C—H⋯Br inter­actions, running along the *c* axis. The solvent di­chloro­methane mol­ecule also makes C—H⋯π and C—H⋯Br inter­actions to the chain. The N—H⋯Br and C—H⋯Br hydrogen bonds are indicated by dashed lines. Hydrogen atoms not involved in the hydrogen bonds are omitted for clarity.

The Br anion is an acceptor of four hydrogen bonds, three of which are donated by phenyl and amine groups of the *trans*-1,2-di­amino­(*N*,*N*′-ditri­phenyl­phospho­nio)cyclo­hexane mol­ecule and the last is donated by the solvent di­chloro­methane mol­ecule (Table 1 ▸). In the hydrogen-bond pattern, the graph-set motif 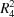
(22) involving atoms (–C19—N1—H1⋯Br1⋯H5—C5—C6—C1—P1—N1—C19–)_2_ can be distinguished (Fig. 2 ▸). The 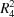
(22) pattern generates a supra­molecular chain running along the *c* axis. The di­chloro­methane mol­ecule is also linked to the chain *via* C—H⋯π and C—H⋯Br inter­actions (Fig. 3 ▸ and Table 1 ▸).

## Database survey   

A search of the Cambridge Structural Database (Version 5.37; Groom & Allen, 2014 ▸) revealed the existence of 33 deposited phospho­nium structures of general formula [*R*_3_PNH*R*′]^+^, where *R* and *R*′ are either aryl or alkyl groups. Amongst those, only two structures are polycationic: MELCIQ (Alajarín *et al.*, 2006 ▸) is tricationic with a tricyclic structure and tri­fluoro­acetate counter-ions, and WERROB (Demange *et al.*, 2006 ▸) is dicationic and contains bromide counter-ions. All the remaining structures are monocationic and only four of them contain a bromide counter-ion: ECUJOC (Boubekeur *et al.*, 2006 ▸), NEPZUF (Martínez de León *et al.*, 2013 ▸), ZOFYAU and ZOFYEY (Imrie *et al.*, 1995 ▸). For all the reported compounds, the P—N bond distances assume a partial double-bond character with values falling within the narrow range of 1.60–1.66 Å, regardless of the counter-ion and substituents on both N and P. The N—P distance of the title compound [1.623 (2) Å] agrees with these values. In addition, the P—N—C angle in the present compound [126.9 (2)°] indicates a planar *sp^2^* geometry for the N atom, and falls within the range of 120–133° reported for all related phospho­nium structures.

## Synthesis and crystallization   

Under an N_2_ atmosphere, a solution of 3.07 g of Br_2_ in 5 ml of CH_2_Cl_2_ was added dropwise under stirring at 273 K, to a solution of Ph_3_P (5.04 g, 19.24 mmol) in 100 ml of the same solvent. After four h of stirring at room temperature and the formation of white precipitate, a mixture of half an equivalent of (±)-*trans*-1,2-di­amino­cyclo­hexane (1.09 g, 9.62 mmol) and one equivalent of tri­ethyl­amine (2.68 ml, 19.24 mmol) in 10 ml of CH_2_Cl_2_ was added dropwise under stirring at 273 K. The suspension was left under continuous stirring for 12 h at room temperature. Then the reactant was extracted twice with 25 ml of distilled water, and the organic phase was dried over MgSO_4_. All volatiles were eliminated under vacuum, and the resulting light-yellow solid was stirred with Et_2_O overnight. After filtration, 6.0 g of the title compound was obtained as a white powder (yield 93%, m.p. 563 K). Single crystals suitable for X-ray diffraction were grown by slow evaporation of a di­chloro­methane solution at room temperature.

## Refinement   

Crystal data, data collection and structure refinement details are summarized in Table 2 ▸. The N-bound H atom was located in a difference Fourier map and its coordinates were refined with a distance restraint of N—H = 0.86 (1) Å with *U*_iso_(H) = 1.2*U*_eq_(N). Other H atoms were positioned geometrically (C—H = 0.93 or 0.97 Å) and constrained using the riding-model approximation with *U*_iso_(H) = 1.2*U*_eq_(C).

## Supplementary Material

Crystal structure: contains datablock(s) Global, I. DOI: 10.1107/S2056989016004576/is5441sup1.cif

Structure factors: contains datablock(s) I. DOI: 10.1107/S2056989016004576/is5441Isup2.hkl

CCDC reference: 1469040

Additional supporting information:  crystallographic information; 3D view; checkCIF report

## Figures and Tables

**Table 1:** Hydrogen-bond geometry (Å, °) *Cg* is the centroid of the C7–C12 ring.

*D*—H⋯*A*	*D*—H	H⋯*A*	*D*⋯*A*	*D*—H⋯*A*
N1—H1⋯Br1	0.86 (3)	2.43 (3)	3.285 (2)	172 (3)
C6—H6⋯Br1^i^	0.93	2.80	3.670 (3)	157
C15—H15⋯Br1^ii^	0.93	2.84	3.718 (3)	158
C22—H22*A*⋯Br1^ii^	0.97	2.80	3.562 (3)	136
C22—H22*B*⋯*Cg* ^ii^	0.97	2.54	3.479	163

Symmetry codes: (i) 
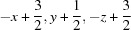
; (ii) 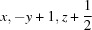.

**Table 2:** Experimental details

Crystal data	
Chemical formula	C_42_H_42_N_2_P_2_^2+^·2Br^−^·2CH_2_Cl_2_
*M*_r_	966.39
Crystal system, space group	Monoclinic, *C*2/*c*
Temperature (K)	100
*a*, *b*, *c* (Å)	17.1911 (2), 14.9027 (2), 18.4492 (2)
β (°)	114.2547 (15)
*V* (Å^3^)	4309.34 (10)
*Z*	4
Radiation type	Cu *K*α
μ (mm^−1^)	5.63
Crystal size (mm)	0.17 × 0.12 × 0.09

Data collection	
Diffractometer	Agilent SuperNova Dual Source diffractometer with an EosS2 detector
Absorption correction	Multi-scan (*CrysAlis PRO*; Agilent, 2014 ▸)
*T*_min_, *T*_max_	0.145, 0.602
No. of measured, independent and observed [*I* > 2σ(*I*)] reflections	16763, 4256, 4203
*R*_int_	0.021
(sin θ/λ)_max_ (Å^−1^)	0.619

Refinement	
*R*[*F* ^2^ > 2σ(*F* ^2^)], *wR*(*F* ^2^), *S*	0.038, 0.098, 1.04
No. of reflections	4256
No. of parameters	248
No. of restraints	1
H-atom treatment	H atoms treated by a mixture of independent and constrained refinement
Δρ_max_, Δρ_min_ (e Å^−3^)	1.54, −1.50

Computer programs: *CrysAlis PRO* (Agilent, 2014 ▸), *SHELXS97* (Sheldrick, 2008 ▸), *SHELXL2014* (Sheldrick, 2015 ▸), *OLEX2* (Dolomanov *et al.*, 2009 ▸), *PLATON* (Spek, 2009 ▸) and *publCIF* (Westrip, 2010 ▸).

## supplementary crystallographic information

### Crystal data

**Table:**

C_42_H_42_N_2_P_2_^2+^·2Br^−^·2CH_2_Cl_2_	*F*(000) = 1968
*M**_r_* = 966.39	*D*_x_ = 1.490 Mg m^−^^3^
Monoclinic, *C*2/*c*	Cu *K*α radiation, λ = 1.54184 Å
*a* = 17.1911 (2) Å	Cell parameters from 13228 reflections
*b* = 14.9027 (2) Å	θ = 2.6–72.5°
*c* = 18.4492 (2) Å	µ = 5.63 mm^−^^1^
β = 114.2547 (15)°	*T* = 100 K
*V* = 4309.34 (10) Å^3^	Plate, colourless
*Z* = 4	0.17 × 0.12 × 0.09 mm

### Data collection

**Table:**

Agilent SuperNova Dual Source diffractometer with an EosS2 detector	4256 independent reflections
Radiation source: sealed X-ray tube, SuperNova (Cu) X-ray Source	4203 reflections with *I* > 2σ(*I*)
Mirror monochromator	*R*_int_ = 0.021
Detector resolution: 8.0769 pixels mm^-1^	θ_max_ = 72.7°, θ_min_ = 4.1°
ω scans	*h* = −20→21
Absorption correction: multi-scan (*CrysAlis PRO*; Agilent, 2014)	*k* = −18→18
*T*_min_ = 0.145, *T*_max_ = 0.602	*l* = −22→22
16763 measured reflections	

### Refinement

**Table:**

Refinement on *F*^2^	Hydrogen site location: mixed
Least-squares matrix: full	H atoms treated by a mixture of independent and constrained refinement
*R*[*F*^2^ > 2σ(*F*^2^)] = 0.038	*w* = 1/[σ^2^(*F*_o_^2^) + (0.041*P*)^2^ + 29.4173*P*] where *P* = (*F*_o_^2^ + 2*F*_c_^2^)/3
*wR*(*F*^2^) = 0.098	(Δ/σ)_max_ = 0.001
*S* = 1.04	Δρ_max_ = 1.54 e Å^−^^3^
4256 reflections	Δρ_min_ = −1.50 e Å^−^^3^
248 parameters	Extinction correction: *SHELXL2014* (Sheldrick, 2015), Fc^*^=kFc[1+0.001xFc^2^λ^3^/sin(2θ)]^-1/4^
1 restraint	Extinction coefficient: 0.00026 (3)

### Special details

**Table:**

Experimental. MS (FAB^+^) 716 m/*z* (M—Br)^+^ 12%; ^31^P NMR (CDCl_3_, 80 MHz, 20°C) 37.05 p.p.m; ^1^H NMR (400 MHz, CDCl_3_, 20°C): δ = 0.909–0.859 (m, 2H, CH_2_), 1.349–1.493 (m, 2H, CH_2_) 1.525–1.493(m, 2H, CH_2_), 1.928–1.902(m, 2H, CH_2_), 3.626–3.615 (m, 2H, CH—N), 7.918–7.864 (m, 12H, *o*-C_6_H_5_), 7.677–7.635 (m, 12H, *m*-C_6_H_5_), 7.553–7.505(m, 6H, *p*-C_6_H_5_), δ 8.59 (s, 2H, NH); ^13^C NMR (100 MHz, CDCl_3_, 20°C): 24.34 (s, 2 C, CH_2_), 36.10 (s, 2 C, CH_2_), 57.949 (dd, ^2^*J*_CP_ = 2.9 Hz, ^3^*J*_CP_ = 10.3 Hz, 2 C, CH—N), 121.372 (d, ^1^*J*_CP_=102.5 Hz, 6 C*ipso*, C_6_H_5_), 129.599 (d, ^3^*J*_CP_ = 13.2 Hz, 12 C*meta*, C_6_H_5_), 134.307 (d, ^4^*J*_CP_ = 2.9 Hz, 6 C*para*, C_6_H_5_,), 134.505 (d, ^2^*J*_CP_ = 7.9 Hz, 12 C*orto*, C_6_H_5_).
Geometry. All e.s.d.'s (except the e.s.d. in the dihedral angle between two l.s. planes) are estimated using the full covariance matrix. The cell e.s.d.'s are taken into account individually in the estimation of e.s.d.'s in distances, angles and torsion angles; correlations between e.s.d.'s in cell parameters are only used when they are defined by crystal symmetry. An approximate (isotropic) treatment of cell e.s.d.'s is used for estimating e.s.d.'s involving l.s. planes.

### Fractional atomic coordinates and isotropic or equivalent isotropic displacement parameters (Å^2^)

**Table:**

	*x*	*y*	*z*	*U*_iso_*/*U*_eq_	
Br1	0.64866 (2)	0.38834 (2)	0.63298 (2)	0.02162 (12)	
P1	0.67384 (4)	0.61073 (4)	0.77021 (4)	0.00659 (15)	
Cl1	0.80208 (4)	0.46666 (4)	1.00873 (4)	0.01662 (16)	
Cl2	0.88621 (4)	0.41641 (5)	1.17643 (4)	0.02132 (17)	
N1	0.61012 (14)	0.52543 (15)	0.75374 (13)	0.0112 (4)	
H1	0.619 (2)	0.4854 (17)	0.7247 (17)	0.013*	
C1	0.78312 (15)	0.57703 (17)	0.82238 (14)	0.0077 (5)	
C2	0.80581 (16)	0.48708 (17)	0.82314 (15)	0.0107 (5)	
H2	0.7643	0.4441	0.7977	0.013*	
C3	0.89130 (17)	0.46215 (19)	0.86238 (16)	0.0146 (5)	
H3	0.9068	0.4022	0.8639	0.017*	
C4	0.95309 (17)	0.5265 (2)	0.89913 (16)	0.0151 (5)	
H4	1.0100	0.5096	0.9248	0.018*	
C5	0.93061 (17)	0.61616 (19)	0.89792 (16)	0.0143 (5)	
H5	0.9726	0.6590	0.9224	0.017*	
C6	0.84553 (16)	0.64224 (18)	0.86028 (15)	0.0105 (5)	
H6	0.8303	0.7021	0.8602	0.013*	
C7	0.66066 (16)	0.66197 (17)	0.67754 (15)	0.0097 (5)	
C8	0.58964 (17)	0.6405 (2)	0.60814 (16)	0.0154 (5)	
H8	0.5494	0.5999	0.6100	0.018*	
C9	0.57900 (18)	0.6797 (2)	0.53601 (16)	0.0190 (6)	
H9	0.5317	0.6655	0.4898	0.023*	
C10	0.63935 (19)	0.7401 (2)	0.53352 (16)	0.0178 (6)	
H10	0.6326	0.7659	0.4854	0.021*	
C11	0.71012 (18)	0.76242 (19)	0.60279 (17)	0.0158 (6)	
H11	0.7501	0.8031	0.6007	0.019*	
C12	0.72098 (17)	0.72393 (18)	0.67497 (16)	0.0123 (5)	
H12	0.7679	0.7391	0.7212	0.015*	
C13	0.64931 (15)	0.69184 (17)	0.82921 (15)	0.0084 (5)	
C14	0.66580 (16)	0.67095 (18)	0.90835 (15)	0.0115 (5)	
H14	0.6924	0.6172	0.9305	0.014*	
C15	0.64235 (18)	0.73048 (19)	0.95346 (16)	0.0143 (5)	
H15	0.6519	0.7162	1.0055	0.017*	
C16	0.60443 (17)	0.81180 (18)	0.92062 (16)	0.0148 (5)	
H16	0.5890	0.8519	0.9510	0.018*	
C17	0.58951 (18)	0.83340 (19)	0.84306 (17)	0.0163 (6)	
H17	0.5647	0.8882	0.8218	0.020*	
C18	0.61150 (17)	0.77332 (18)	0.79696 (16)	0.0134 (5)	
H18	0.6009	0.7876	0.7447	0.016*	
C19	0.54401 (15)	0.51184 (17)	0.78478 (14)	0.0089 (5)	
H19	0.5465	0.5627	0.8194	0.011*	
C20	0.55975 (19)	0.4261 (2)	0.83400 (18)	0.0231 (7)	
H20A	0.6181	0.4261	0.8739	0.028*	
H20B	0.5222	0.4253	0.8614	0.028*	
C21	0.5447 (2)	0.3415 (2)	0.7836 (3)	0.0352 (9)	
H21A	0.5515	0.2891	0.8168	0.042*	
H21B	0.5868	0.3382	0.7612	0.042*	
C22	0.79291 (18)	0.4616 (2)	1.10118 (17)	0.0178 (6)	
H22A	0.7831	0.5215	1.1164	0.021*	
H22B	0.7443	0.4247	1.0955	0.021*	

### Atomic displacement parameters (Å^2^)

**Table:**

	*U*^11^	*U*^22^	*U*^33^	*U*^12^	*U*^13^	*U*^23^
Br1	0.02498 (19)	0.01596 (17)	0.0295 (2)	−0.00524 (11)	0.01685 (14)	−0.00967 (12)
P1	0.0049 (3)	0.0080 (3)	0.0064 (3)	−0.0004 (2)	0.0019 (2)	−0.0009 (2)
Cl1	0.0175 (3)	0.0173 (3)	0.0136 (3)	0.0011 (2)	0.0049 (2)	0.0002 (2)
Cl2	0.0207 (3)	0.0256 (4)	0.0166 (3)	0.0007 (3)	0.0065 (3)	0.0065 (3)
N1	0.0089 (10)	0.0127 (11)	0.0148 (11)	−0.0054 (8)	0.0075 (9)	−0.0065 (9)
C1	0.0056 (11)	0.0111 (12)	0.0059 (11)	0.0000 (9)	0.0018 (9)	0.0011 (9)
C2	0.0104 (12)	0.0108 (12)	0.0103 (12)	−0.0009 (10)	0.0038 (10)	−0.0004 (9)
C3	0.0146 (13)	0.0147 (13)	0.0142 (13)	0.0049 (10)	0.0058 (11)	0.0021 (10)
C4	0.0094 (12)	0.0252 (15)	0.0102 (12)	0.0029 (11)	0.0036 (10)	0.0026 (11)
C5	0.0087 (12)	0.0212 (14)	0.0106 (12)	−0.0057 (10)	0.0014 (10)	−0.0026 (10)
C6	0.0111 (12)	0.0101 (12)	0.0095 (11)	−0.0016 (10)	0.0033 (10)	−0.0013 (9)
C7	0.0111 (12)	0.0117 (12)	0.0071 (11)	0.0043 (10)	0.0046 (10)	0.0010 (9)
C8	0.0086 (12)	0.0236 (14)	0.0127 (13)	−0.0004 (11)	0.0032 (10)	0.0002 (11)
C9	0.0149 (13)	0.0284 (16)	0.0094 (12)	0.0038 (12)	0.0006 (11)	0.0017 (11)
C10	0.0229 (15)	0.0201 (14)	0.0100 (12)	0.0072 (11)	0.0064 (11)	0.0052 (11)
C11	0.0198 (14)	0.0129 (13)	0.0160 (13)	0.0015 (11)	0.0087 (11)	0.0031 (10)
C12	0.0132 (12)	0.0114 (12)	0.0111 (12)	0.0011 (10)	0.0037 (10)	0.0007 (10)
C13	0.0063 (11)	0.0092 (11)	0.0103 (11)	−0.0017 (9)	0.0038 (9)	−0.0027 (9)
C14	0.0122 (12)	0.0108 (12)	0.0096 (12)	−0.0003 (10)	0.0024 (10)	0.0000 (10)
C15	0.0173 (13)	0.0165 (13)	0.0089 (12)	−0.0029 (11)	0.0051 (10)	−0.0027 (10)
C16	0.0161 (13)	0.0132 (13)	0.0162 (13)	−0.0019 (10)	0.0078 (11)	−0.0073 (10)
C17	0.0193 (14)	0.0101 (12)	0.0200 (14)	0.0037 (10)	0.0084 (11)	0.0001 (11)
C18	0.0152 (13)	0.0138 (13)	0.0119 (12)	0.0028 (10)	0.0062 (10)	0.0028 (10)
C19	0.0068 (12)	0.0125 (12)	0.0085 (11)	−0.0028 (9)	0.0042 (10)	−0.0022 (9)
C20	0.0123 (13)	0.0358 (18)	0.0209 (14)	0.0096 (12)	0.0067 (11)	0.0199 (13)
C21	0.043 (2)	0.0136 (15)	0.070 (3)	0.0149 (14)	0.043 (2)	0.0217 (16)
C22	0.0172 (14)	0.0196 (14)	0.0205 (14)	0.0039 (11)	0.0118 (12)	0.0039 (11)

### Geometric parameters (Å, º)

**Table:**

P1—C13	1.789 (3)	C11—C10	1.395 (4)
P1—N1	1.623 (2)	C12—H12	0.9300
P1—C7	1.800 (3)	C12—C11	1.390 (4)
P1—C1	1.795 (2)	C12—C7	1.404 (4)
Cl1—C22	1.778 (3)	C13—C14	1.403 (4)
Cl2—C22	1.767 (3)	C13—C18	1.390 (4)
N1—H1	0.856 (10)	C14—H14	0.9300
N1—C19	1.482 (3)	C14—C15	1.385 (4)
C2—H2	0.9300	C15—H15	0.9300
C2—C1	1.395 (4)	C16—H16	0.9300
C3—H3	0.9300	C16—C17	1.384 (4)
C3—C2	1.396 (4)	C16—C15	1.392 (4)
C4—H4	0.9300	C17—H17	0.9300
C4—C3	1.385 (4)	C18—H18	0.9300
C5—H5	0.9300	C18—C17	1.390 (4)
C5—C4	1.388 (4)	C19—C19^i^	1.530 (5)
C5—C6	1.392 (4)	C19—H19	0.9800
C6—H6	0.9300	C19—C20	1.526 (4)
C6—C1	1.403 (3)	C20—H20A	0.9700
C8—H8	0.9300	C20—H20B	0.9700
C8—C7	1.396 (4)	C21—C21^i^	1.527 (8)
C8—C9	1.395 (4)	C21—H21A	0.9700
C9—H9	0.9300	C21—H21B	0.9700
C10—H10	0.9300	C21—C20	1.524 (5)
C10—C9	1.389 (4)	C22—H22A	0.9700
C11—H11	0.9300	C22—H22B	0.9700
			
C13—P1—C7	108.80 (12)	C7—C12—H12	120.2
C13—P1—C1	108.61 (12)	C14—C13—P1	119.1 (2)
N1—P1—C13	109.47 (12)	C18—C13—P1	121.0 (2)
N1—P1—C7	110.03 (12)	C18—C13—C14	119.9 (2)
N1—P1—C1	111.00 (12)	C13—C14—H14	120.0
C1—P1—C7	108.89 (12)	C15—C14—C13	119.9 (2)
P1—N1—H1	113 (2)	C15—C14—H14	120.0
C19—N1—P1	126.91 (18)	C14—C15—C16	119.7 (2)
C19—N1—H1	121 (2)	C14—C15—H15	120.1
C6—C1—P1	119.42 (19)	C16—C15—H15	120.1
C2—C1—P1	120.07 (19)	C17—C16—H16	119.8
C2—C1—C6	120.5 (2)	C17—C16—C15	120.5 (2)
C3—C2—H2	120.3	C15—C16—H16	119.8
C1—C2—C3	119.5 (2)	C16—C17—C18	120.1 (3)
C1—C2—H2	120.3	C16—C17—H17	120.0
C4—C3—H3	119.9	C18—C17—H17	120.0
C4—C3—C2	120.1 (3)	C13—C18—H18	120.1
C2—C3—H3	119.9	C17—C18—C13	119.8 (2)
C5—C4—H4	119.8	C17—C18—H18	120.1
C3—C4—C5	120.4 (2)	N1—C19—C19^i^	109.1 (2)
C3—C4—H4	119.8	N1—C19—H19	108.1
C6—C5—H5	119.8	N1—C19—C20	111.6 (2)
C4—C5—H5	119.8	C19^i^—C19—H19	108.1
C4—C5—C6	120.3 (2)	C20—C19—C19^i^	111.81 (18)
C5—C6—H6	120.4	C20—C19—H19	108.1
C5—C6—C1	119.1 (2)	C19—C20—H20A	109.1
C1—C6—H6	120.4	C19—C20—H20B	109.1
C12—C7—P1	120.3 (2)	C21—C20—C19	112.6 (3)
C8—C7—P1	119.7 (2)	C21—C20—H20A	109.1
C8—C7—C12	120.0 (2)	C21—C20—H20B	109.1
C7—C8—H8	120.0	H20A—C20—H20B	107.8
C9—C8—H8	120.0	C21^i^—C21—H21A	109.5
C9—C8—C7	120.1 (3)	C21^i^—C21—H21B	109.5
C8—C9—H9	120.1	H21A—C21—H21B	108.0
C10—C9—C8	119.8 (3)	C20—C21—C21^i^	110.9 (2)
C10—C9—H9	120.1	C20—C21—H21A	109.5
C11—C10—H10	119.8	C20—C21—H21B	109.5
C9—C10—C11	120.4 (3)	Cl1—C22—H22A	109.4
C9—C10—H10	119.8	Cl1—C22—H22B	109.4
C12—C11—H11	119.9	Cl2—C22—Cl1	111.12 (15)
C12—C11—C10	120.2 (3)	Cl2—C22—H22A	109.4
C10—C11—H11	119.9	Cl2—C22—H22B	109.4
C11—C12—H12	120.2	H22A—C22—H22B	108.0
C11—C12—C7	119.6 (2)		
			
P1—C13—C14—C15	−176.0 (2)	C7—P1—C13—C18	10.7 (2)
P1—C13—C18—C17	177.0 (2)	C7—P1—N1—C19	−122.4 (2)
P1—N1—C19—C19^i^	115.00 (18)	C7—P1—C1—C6	75.4 (2)
P1—N1—C19—C20	−121.0 (2)	C7—P1—C1—C2	−102.7 (2)
N1—P1—C13—C14	68.0 (2)	C7—C12—C11—C10	0.5 (4)
N1—P1—C13—C18	−109.6 (2)	C7—C8—C9—C10	0.1 (4)
N1—P1—C7—C12	−166.0 (2)	C9—C8—C7—P1	−179.9 (2)
N1—P1—C7—C8	14.6 (3)	C9—C8—C7—C12	0.7 (4)
N1—P1—C1—C6	−163.30 (19)	C11—C12—C7—P1	179.6 (2)
N1—P1—C1—C2	18.5 (2)	C11—C12—C7—C8	−1.0 (4)
N1—C19—C20—C21	−69.6 (3)	C11—C10—C9—C8	−0.6 (4)
C1—P1—C13—C14	−53.3 (2)	C12—C11—C10—C9	0.3 (4)
C1—P1—C13—C18	129.0 (2)	C13—P1—N1—C19	−2.9 (3)
C1—P1—N1—C19	117.0 (2)	C13—P1—C7—C12	74.1 (2)
C1—P1—C7—C12	−44.1 (2)	C13—P1—C7—C8	−105.4 (2)
C1—P1—C7—C8	136.4 (2)	C13—P1—C1—C6	−42.9 (2)
C3—C2—C1—P1	178.5 (2)	C13—P1—C1—C2	138.9 (2)
C3—C2—C1—C6	0.4 (4)	C13—C14—C15—C16	−1.6 (4)
C4—C5—C6—C1	−1.2 (4)	C13—C18—C17—C16	−0.6 (4)
C4—C3—C2—C1	−1.0 (4)	C14—C13—C18—C17	−0.6 (4)
C5—C6—C1—P1	−177.4 (2)	C15—C16—C17—C18	0.7 (4)
C5—C6—C1—C2	0.7 (4)	C17—C16—C15—C14	0.4 (4)
C5—C4—C3—C2	0.6 (4)	C18—C13—C14—C15	1.7 (4)
C6—C5—C4—C3	0.5 (4)	C19^i^—C19—C20—C21	52.9 (3)
C7—P1—C13—C14	−171.7 (2)	C21^i^—C21—C20—C19	−55.1 (4)

Symmetry code: (i) −*x*+1, *y*, −*z*+3/2.

### Hydrogen-bond geometry (Å, º)

*Cg* is the centroid of the C7–C12 ring.

**Table:**

*D*—H···*A*	*D*—H	H···*A*	*D*···*A*	*D*—H···*A*
N1—H1···Br1	0.86 (3)	2.43 (3)	3.285 (2)	172 (3)
C6—H6···Br1^ii^	0.93	2.80	3.670 (3)	157
C15—H15···Br1^iii^	0.93	2.84	3.718 (3)	158
C22—H22*A*···Br1^iii^	0.97	2.80	3.562 (3)	136
C22—H22*B*···*Cg*^iii^	0.97	2.54	3.479	163

Symmetry codes: (ii) −*x*+3/2, *y*+1/2, −*z*+3/2; (iii) *x*, −*y*+1, *z*+1/2.
